# From high-salt diet to cancer immunity: gut microbiota and immune system interactions and their mechanisms

**DOI:** 10.3389/fimmu.2026.1948343

**Published:** 2026-10-02

**Authors:** Jiayu Dong, Liyong Zhou

**Affiliations:** Sichuan Academy of Medical Sciences and Sichuan Provincial People’s Hospital, Chengdu, China

**Keywords:** cancer immunity, gut microbiota, high-salt diet, inflammatory response, microbial metabolites

## Abstract

High-salt diet (HSD) has traditionally been studied in the context of cardiovascular disease, hypertension, and kidney disease. Increasing evidence now suggests that salt intake may also influence cancer immunity through direct effects on immune cells and indirect effects mediated by the gut microbiota. HSD can regulate inflammatory activation in T cells, macrophages, NK cells, and other immune populations. It can also reshape gut microbial composition and alter microbial metabolites, including short-chain fatty acids (SCFAs), bile acids (BAs), and tryptophan (Trp) metabolites. These changes may affect immune homeostasis, inflammatory signaling, and the tumor microenvironment. Current evidence suggests that the HSD-microbiota-immune axis has context-dependent roles in cancer biology. In some experimental settings, HSD may enhance antitumor immune responses, whereas chronic exposure may promote inflammation, immune exhaustion, microbial dysbiosis, and tumor progression. In this review, we summarize how HSD interacts with the gut microbiota and immune system, discuss its potential bidirectional effects on cancer initiation and progression, and highlight future directions for diet-microbiota-immune interventions in cancer prevention and therapy.

## Introduction

Dietary salt is essential for normal physiological functions, particularly for maintaining osmotic pressure and electrolyte balance across cells. However, when intake exceeds physiological needs, excessive salt consumption can have substantial adverse effects on health (1). According to the World Health Organization, the global average salt intake is approximately 10.8 g per day, more than twice the recommended daily intake of no more than 5 g. With the widespread adoption of high-salt dietary patterns, the health risks of excessive salt intake have become increasingly evident, particularly in cardiovascular disease, hypertension, and kidney disease (2, 3). More recently, attention has shifted toward the effects of HSD on the immune system, especially its potential roles in regulating gut microbiota, immune-cell differentiation and activation, and cancer immunity.

The gut microbiota plays an important role in host metabolism, and disruption of microbial homeostasis is closely associated with the development of multiple cancers. Increasing evidence indicates that gut microbiota dysbiosis not only alters host immune responses but also promotes tumor initiation and progression through complex immune and metabolic networks. HSD has been shown to markedly affect the composition and function of the gut microbiota. Through these microbial changes, HSD may indirectly regulate immune-cell activity, immune responses, and tumor immune escape. Experimental studies further indicate that HSD can modulate immune-cell function in the spleen, gut-associated lymphoid tissue (GALT), and central nervous system by altering gut microbial structure and metabolites, including SCFAs (4–8).

The gut microbiota also influences exposure to potentially carcinogenic metabolites by modifying dietary components, and it has important effects on immune responses in cancer (9–11). Microbial metabolites such as SCFAs have been implicated in multiple inflammatory diseases and cancers. By regulating immune-cell differentiation and function, gut microbiota changes may therefore play complex roles in cancer immunity. In tumor immunotherapy, modulation of the gut microbiota has become an important research focus, and HSD-induced microbiota changes may have dual effects on immune escape and cancer progression.

Although preliminary evidence suggests that HSD may stimulate immune responses and suppress tumor growth by promoting a proinflammatory environment, its overall effects remain context-dependent. Further studies are needed to distinguish the direct effects of HSD on immune cells from its indirect effects mediated by the gut microbiota. Current evidence indicates that HSD may act through direct immune-cell activation, including modulation of CD4^+^ and CD8^+^ T-cell effector function. It may also influence tumor immune escape by reshaping the gut microbiota and altering the immune milieu. Understanding the interactions among high salt, the gut microbiota, and the immune system may help clarify how HSD affects cancer development and treatment across different immune microenvironments (4). Here, we focus on the potential effects of high salt-microbiota-immune interactions on cancer and propose directions for future research (**Figure 1**). Overall, this review helps clarify the complex relationships within the HSD-gut microbiota-immune axis and provides insights for developing microbiota-informed strategies for cancer prevention and therapy.

**Figure 1:**
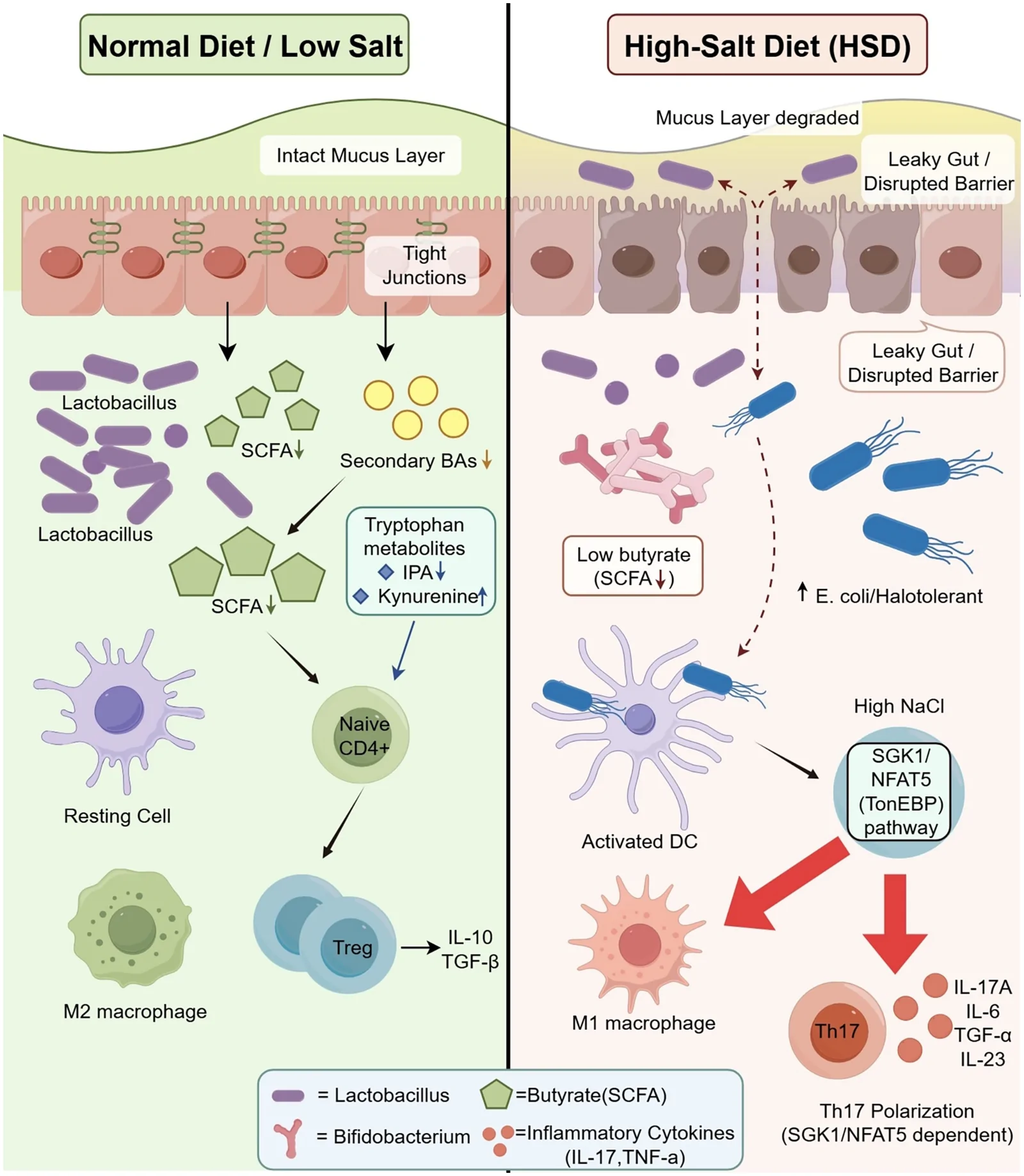
Conceptual overview of the HSD-microbiota-immune-cancer axis. High-salt diet (HSD) may affect cancer immunity through two interconnected mechanisms: a direct microbiota-independent pathway, in which increased Na+ exposure and hyperosmotic stress regulate immune-cell signaling and function, and a microbiota-dependent pathway, in which HSD reshapes gut microbial composition and microbial metabolites. These pathways converge on cancer immunoediting, thereby influencing immune surveillance, immune equilibrium, immune exhaustion, and immune escape.

### Direct microbiota-independent effects of HSD on cancer immunity

Elevated extracellular Na+ concentrations can directly affect immune cells by increasing local osmotic pressure and activating osmotic-stress-responsive signaling pathways. Immune cells exposed to hyperosmotic conditions may activate transcriptional and kinase pathways such as NFAT5, SGK1, and p38/MAPK, which regulate cytokine production, T-cell differentiation, and effector-cell metabolism. In T cells, these pathways may enhance glycolytic activity, mitochondrial function, and the production of effector molecules required for cytotoxic responses. Therefore, under certain experimental conditions, high-salt or Na+-enriched environments may enhance antitumor immune activity by promoting T-cell activation, IFN-γ production, and cytotoxic function. However, this effect should be interpreted as context-dependent, because prolonged or chronic HSD exposure may instead promote persistent inflammation, Treg dysfunction, immune exhaustion, and tumor immune escape.

### Antitumor immune activation under high-salt conditions

HSD is considered a potent immunomodulatory factor that can activate multiple immune-cell populations, particularly T cells and macrophages, and thereby promote local inflammatory responses (10). These cells initiate inflammation by secreting proinflammatory cytokines, including IL-6 and TNF-α. By increasing tissue sodium concentrations, HSD alters osmotic pressure and salinity within the immune microenvironment, which can affect immune-cell function and activity (12). This effect may be particularly relevant in the tumor microenvironment (TME), where salt-mediated changes in T-cell activation and macrophage differentiation can reshape antitumor immunity and influence tumor initiation or progression.

Experimental evidence suggests that CD4^+^ T cells expanded *in vitro* under high-salt conditions retain tumor specificity and show enhanced cytotoxicity against Py230 breast cancer cells, a mouse model commonly used in breast cancer immunotherapy research (4). These CD4^+^ T cells significantly inhibited syngeneic tumor growth, indicating that high-salt-expanded CD4^+^ T cells may have antitumor potential. This shift toward an effector phenotype is closely linked to changes in glycolysis and mitochondrial activity. After high-salt treatment, metabolic remodeling may enhance CD4^+^ T-cell antitumor activity by supporting the synthesis of cytokines, metabolic enzymes, and other molecules required for effector responses (13).

Recent experimental studies further indicate that sodium chloride (NaCl) can reverse T-cell dysfunction and restore antitumor immune responses by regulating immunometabolism. In CD8^+^ T-cell culture, high salt promotes human CD8^+^ T-cell differentiation, IFN-γ production, and cytotoxicity, while maintaining gene networks associated with stem-like plasticity. These transcriptional programs may help preserve long-term immune memory and antitumor capacity (10).

### Chronic inflammation, immune dysfunction, and tumor-promoting effects

Although some studies suggest that HSD may have potential benefits in cancer treatment, recommending it as a routine dietary strategy is not realistic. High salt intake can also promote cancer progression by activating proinflammatory immune responses, which may support sustained cell proliferation, DNA damage, and tumor transformation (14). In particular, salt can activate immune cells such as T cells and macrophages, stimulate the production of inflammatory cytokines including IL-6 and TNF-α, and thereby create conditions that favor tumor growth.

One important mechanism involves the Th17/IL-17 axis. Evidence indicates that high salt intake can induce Th17-cell differentiation, with IL-17A as a major Th17-derived cytokine. Excessive IL-17A production can trigger local proinflammatory responses and further promote tumor progression by activating tumor-associated macrophages and other immune cells (15). In breast cancer models, IL-17A has been shown to enhance tumor-cell proliferation and migration and to promote immune escape in the TME. HSD may also promote breast cancer progression and lung metastasis by increasing the proportion of Th17 cells and stimulating IL-17F secretion. IL-17F can activate MAPK/ERK signaling in breast cancer cells, thereby accelerating proliferation, migration, and invasion. This process may drive tumor growth and dissemination by regulating cell-cycle activity and altering immune-cell function in the TME (16).

Other immune-cell populations may also be affected by chronic salt exposure. Recent animal studies found that chronic HSD increases the proportion of tumor-infiltrating immune cells (TISCs) in the TME, suppresses cytotoxic T-cell function, and weakens adaptive immune responses (17). This attenuation of antitumor immunity may allow tumor cells to evade immune surveillance. In mouse models, HSD has also been shown to impair NK-cell development and function by altering cellular metabolism and reducing receptor expression (18). Such effects may reduce the contribution of NK cells to tumor immune surveillance and promote immune escape. In addition, high salt may exert genotoxic effects. High-salt exposure can impair DNA repair by increasing oxidative stress (ROS), thereby promoting tumor-cell growth and aggravating mutation accumulation and tumor progression (19).

HSD may also increase gastric cancer risk through H. pylori-associated mechanisms (19–21). Under HSD conditions, the high-salt gastric environment can alter H. pylori gene expression, particularly cagA expression, thereby promoting inflammation and enhancing carcinogenic effects (22). In animal experiments, increased dietary salt significantly changed the infection outcomes of cagA^+^ H. pylori strains. High salt enhanced cagA expression, aggravated gastric inflammation, reduced gastric acid secretion, and strengthened carcinogenic effects (21). These changes may intensify local immune responses and promote gastric mucosal injury and malignant transformation. Another model proposes that high salt alters the expression of multiple H. pylori genes, including cagA, thereby changing host immune responses and promoting gastric inflammation (23). HSD can further promote chronic gastric mucosal inflammation by enhancing H. pylori colonization. Persistent H. pylori-driven inflammation reshapes the gastric immune microenvironment and creates favorable conditions for malignant transformation (24). Salt may also support tumor-cell survival and dissemination by regulating host immune responses and cytokine secretion. In gastric epithelial cells, salt stimulation can increase cell survival by activating proliferative pathways such as MAPK/ERK signaling and inhibiting apoptosis (25, 26). These cellular changes may provide a growth advantage during gastric carcinogenesis.

### Tumor-type and stage-dependent effects of HSD

Although the relationship between HSD and cancer remains under active investigation, current evidence suggests that HSD may exert context-dependent effects on tumor progression, ranging from immune-mediated tumor clearance to inflammation-driven tumor promotion (**Figure 2**). During early tumor development, high salt may activate immune responses and support the initiation of antitumor immune surveillance. However, during prolonged exposure or advanced tumor progression, HSD may contribute to immune dysfunction and exhaustion, thereby facilitating tumor immune escape and growth. These effects may vary according to tumor type, disease stage, and the metabolic and immune features of the tumor microenvironment. For example, one animal study found that HSD had no significant effect on acute myeloid leukemia (AML) progression, suggesting that salt-related effects are tumor-specific and may not apply uniformly across cancer types (27). Thus, differences in the immune microenvironment and metabolic requirements of different tumors may explain why distinct tumor contexts respond differently to HSD. **Table 1** synthesizes experimental and mechanistic evidence linking high-salt or sodium exposure to cancer-related outcomes. The evidence includes H. pylori-associated gastric carcinogenesis and bacterial virulence (21–23, 27), breast cancer models involving IL-17 signaling, sodium channels, angiogenesis, metabolic reprogramming, and drug resistance (28–32), and immune-mediated tumor control or tumor-specific responses involving CD4^+^ T cells, CD8^+^ T cells, MDSCs, NK cells, genotoxic stress, and context-dependent melanoma or colorectal cancer mechanisms (7, 8, 10, 16–18, 33–40). **Table 2** summarizes population and systematic-review evidence linking salt or sodium intake with gastric, esophageal, and renal cancer risk (19, 24, 41–46). Together, these findings indicate that the impact of HSD on cancer should be interpreted as a context-dependent process shaped by tumor type, exposure duration, immune status, and microbiota-related mechanisms.

**Figure 2:**
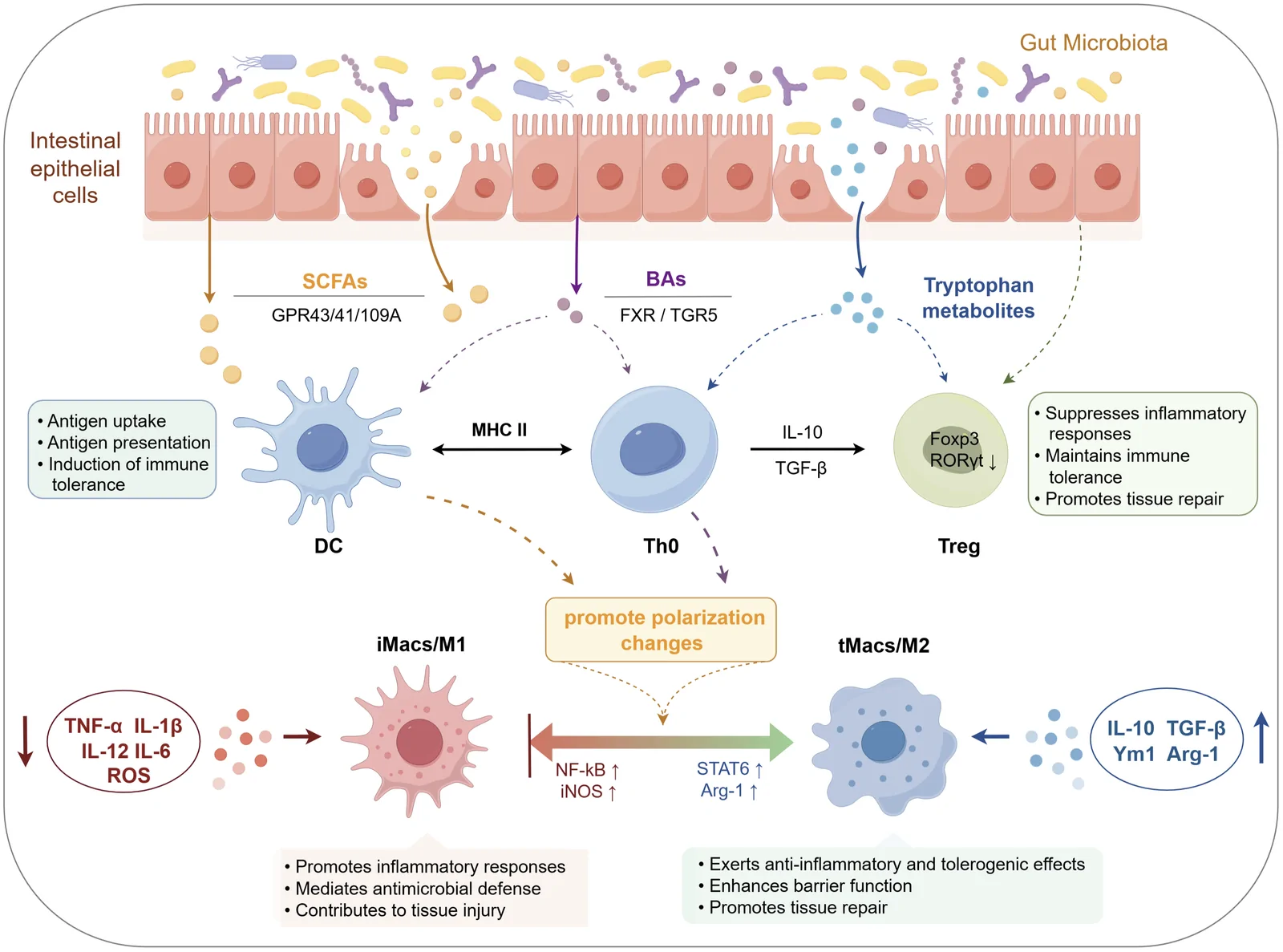
Context-dependent dual effects of HSD on cancer immunity HSD/Na+ exposure may exert both antitumor immune effects and pro-tumor or immune-escape effects depending on tumor type, exposure duration, immune status, and the tumor microenvironment. In certain experimental settings, HSD may enhance T-cell activation, cytotoxicity, metabolic remodeling, and tumor-cell clearance. In contrast, chronic HSD exposure may promote persistent inflammation, immune dysfunction, checkpoint-associated immune exhaustion, tumor immune escape, and tumor progression. Context-dependent Th17/IL-17 signaling may contribute to either antitumor immunity or tumor-promoting inflammation in specific settings.

**Table 1:** Experimental and mechanistic studies on the relationship between high-salt/sodium exposure and cancer.

Reference	Model	Salt treatment/dose	Cancer type	Direction of effect	Microbiota/immunity involved	Mechanistic summary
(27)	Mongolian gerbils; H. pylori infection plus chemical carcinogenesis model	Dose-gradient HSD (2.5%, 5%, and 10% NaCl)	Gastric cancer	Pro-tumorigenic/dose-dependent	H. pylori and inflammation involved	HSD dose-dependently promoted gastric chemical carcinogenesis in the setting of H. pylori infection, indicating that the strength of gastric cancer risk is related to salt intake.
(21)	Mongolian gerbils infected with WT or cagA-mutant H. pylori	Regular chow vs additional 8% NaCl high-salt chow; 4 months after infection	Gastric adenocarcinoma	Pro-tumorigenic/synergistic	H. pylori and inflammation involved	High salt aggravated gastritis, increased gastric pH, and enhanced the carcinogenic effect of cagA^+^ H. pylori; gastric adenocarcinoma incidence was higher in the WT infection plus high-salt group than in the WT regular-diet group, whereas cagA-mutant strains did not show the same carcinogenic effect.
(23)	*In vitro* mechanistic study of H. pylori	Increased salt concentration in the culture environment	Gastric cancer-related bacterial virulence mechanism	Mechanistic support	H. pylori involved	The study identified promoter elements in cagA that mediate salt-induced upregulation of CagA expression, providing a mechanistic basis for high salt to enhance H. pylori carcinogenic virulence.
(22)	*In vitro* H. pylori proteomics	Altered salt concentration in the culture environment	Gastric cancer-related bacterial virulence mechanism	Mechanistic support	H. pylori involved	Changes in salt concentration altered the H. pylori membrane proteome, suggesting that high salt may affect gastric cancer risk by modulating bacterial adaptation and virulence-related proteins.
(28)	Breast cancer cells; high salt plus IL-17 inflammatory model	Additional 0.05 M NaCl (50 mM NaCl) with or without low-dose IL-17	Breast cancer	Pro-tumorigenic/mechanistic support	Related to the immune factor IL-17	γENaC participated in high salt- and IL-17-induced inflammatory stress in breast cancer cells, suggesting that sodium channels may connect the salt environment with tumor-associated inflammation.
(29)	Breast cancer cells; high salt plus IL-17 model	High salt (0.2 M NaCl) plus low-dose IL-17 (1 ng/ml)	Breast cancer	Pro-tumorigenic/angiogenic	Related to the immune factor IL-17	High salt and IL-17 promoted VEGF-A expression through the NFAT5/STAT3 axis, suggesting that salt-associated inflammation may increase the angiogenic potential of tumors.
(30)	Breast cancer cells; osmotic/high-salt stress	High-salt osmotic stress (0.16 M NaCl)	Breast cancer	Pro-tumorigenic/metabolic reprogramming	No, or indirect inflammation	High salt induced a Warburg-like metabolic effect; oleanolic acid inhibited this metabolic change, suggesting that the salt environment can influence tumor metabolism.
(31)	Breast cancer cells; phosphoproteomics	Additional 50 mM NaCl plus IL-17 0.1 ng/mL	Breast cancer	Pro-tumorigenic	Related to the immune factor IL-17	High salt and low-dose IL-17 synergistically induced breast cancer cell proliferation; SIK3, mTORC2, HDAC4, CXCR4, and related pathways were involved.
(32)	Breast cancer cells and xenografts in immunodeficient mice	*In vitro* pretreatment with additional 0.05 M NaCl, followed by *in vivo* tumor formation assessment	Breast cancer/therapeutic tolerance	Pro-tumorigenic/drug resistance	No; intracellular calcium signaling	High salt induced P-glycoprotein-associated therapeutic resistance and increased tumor-cell tumorigenicity through store-operated calcium entry.
(16)	Mouse breast cancer model; analysis of tumor-infiltrating immune cells and tumor-initiating stem-like cells	4% NaCl high-salt chow plus drinking water containing 1% sodium chloride (6 weeks)	Breast cancer	Pro-tumorigenic	Immune-related; TISC/T-cell function	Chronic HSD increased the proportion of TISCs in the tumor microenvironment, suppressed cytotoxic T-cell function, and promoted breast cancer proliferation.
(10)	*In vitro* high-salt expansion of tumor-primed CD4^+^ T cells; Py230 breast cancer transplantation	Additional 40 mM NaCl in T-cell culture	Breast cancer	Anti-tumor/immunotherapeutic potential	Immune-related; CD4^+^ T cells	Tumor-primed CD4^+^ T cells activated ex vivo under high-salt conditions retained tumor specificity and showed enhanced anti-tumor activity, suppressing syngeneic tumor growth after adoptive transfer.
(33)	Mouse transplanted tumor models; *in vitro* and *in vivo* MDSC analysis	HSD animal experiments: 4% NaCl high-salt chow plus drinking water containing 1% sodium chloride (4 weeks); *in vitro* MDSCs treated with additional 40 mM NaCl	Melanoma/lung cancer and other transplanted tumors	Anti-tumor	Immune-related; MDSCs	High salt inhibited the immunosuppressive function of MDSCs and enhanced anti-tumor immunity, thereby slowing tumor growth.
(7)	B16F10 melanoma and 4T1 breast cancer mice; combination with anti-PD-1	NSD 0.4% NaCl chow plus water; HSD 4% NaCl chow plus 1% NaCl drinking water for 16 days	Melanoma/breast cancer	Anti-tumor/enhanced immunotherapy	Immune-related; MDSCs	HSD promoted p38/MAPK-NFAT5-related differentiation of M-MDSCs into anti-tumor-like macrophages and improved anti-PD-1 efficacy.
(8)	Mouse tumor models; antibiotic, FMT, and microbiota-metabolite validation	Animal experiments (ND 0.9%NaCl; LSD 1.0%NaCl; HSD 4%NaCl) for 18–20 days	Solid tumors/tumor immunity	Anti-tumor	Microbiota plus immunity; NK cells	HSD enhanced tumor immunity through gut microbiota-NK cell interactions, accompanied by changes in Bifidobacterium and hippurate, increased NK-cell IFN-γ, and reduced PD-1.
(34)	Two syngeneic AML transplantation mouse models	HSD animal experiment (HSD 4%NaCl) for 3–4 weeks	Acute myeloid leukemia	No obvious effect	Microbiota/immunity measured but no tumor effect	HSD altered the microbiota, Th17 responses, and NK-cell-related markers but did not affect AML initiation or progression, suggesting tumor-type specificity in salt-cancer effects.
(17)	Mice; study of NK-cell development and function	HSD animal experiment (HSD 4% NaCl chow plus 1% NaCl drinking water for 2 months)	Tumor-immunity mechanism	Potential pro-tumor risk	Immune-related; NK cells	HSD disrupted NK-cell development and function and reduced NK-cell maturation and receptor expression, which may weaken tumor immune surveillance.
(35)	Human and mouse CD8^+^ T cells; cancer immunotherapy models	NaCl supplementation in CD8^+^ T-cell culture (approximately additional 30 mM NaCl)	Cancer immunotherapy	Anti-tumor/sensitizing	Immune-related; CD8^+^ T cells	NaCl reversed T-cell dysfunction and promoted IFN-γ production, cytotoxicity, and effector differentiation while maintaining stem-like plasticity gene networks.
(36)	Human CD8^+^ T cells and CAR-T cells; PancOVA and other mouse tumor models; sodium imaging of human breast cancer samples	Approximately additional 30 mM NaCl *in vitro*; sodium enrichment in the solid-tumor microenvironment	Solid tumors/immunotherapy	Anti-tumor/enhanced T-cell killing	Immune-related; CD8^+^/CAR-T	Sodium enrichment in tumor tissues enhanced CD8^+^ T-cell metabolic adaptation and cytotoxicity; high-salt pretreatment of T cells/CAR-T cells improved tumor control.
(37)	AOM/DSS plus ETBF-infected mouse colon cancer model	4% and 8% NaCl high-salt diets	Colorectal cancer	Anti-tumor/context-dependent	Microbiota plus inflammation; ETBF/IL-17A/iNOS	Contrary to the conventional pro-inflammatory expectation, HSD reduced ETBF-promoted colonic tumorigenesis and was accompanied by reduced IL-17A and iNOS, suggesting that HSD effects may be reversed under specific bacterial-strain or inflammatory contexts.
(38)	Orthotopic CT26 colorectal cancer mice; FOLFOX treatment; comparison of microbiota metabolism between patients with CRC and healthy controls	8% NaCl HSD	Colorectal cancer/chemotherapy efficacy	Reduced treatment efficacy	Microbiota plus metabolism plus macrophages	HSD reduced FOLFOX efficacy; gut microbial Trp metabolites, such as SK and IPA, regulated chemotherapy responses through macrophages.
(18)	Cell/tissue hyperosmotic high-salt stress model	High-NaCl exposure	Cancer-related genotoxic mechanism	Mechanistic support	No	High NaCl induced DNA double-strand breaks and affected DNA damage responses, providing non-tumor-model mechanistic evidence that chronic high salt may increase mutational or carcinogenic risk.
(39)	Orthotopic and metastatic 4T1 mouse breast cancer model	1%, 2%, and 4% NaCl high-salt diets for 5 weeks	Breast cancer and lung metastasis	Inhibited primary tumors and lung metastasis	No	4% NaCl inhibited primary tumors and lung metastasis, potentially in part by mimicking caloric restriction; it reduced Ki-67, increased p53, and induced p53-dependent anoikis through hyperosmotic stress.
(40)	C57BL/6J mouse B16-OVA melanoma subcutaneous/lung metastasis model; Y-1 adrenocortical tumor control	NSD 0.5% NaCl; HSD 4% NaCl chow plus 0.9% NaCl drinking water, usually starting 7 days before tumor inoculation	Melanoma; adrenocortical tumor control	Inhibited melanoma, with tumor-type differences	No	HSD-mediated melanoma inhibition was independent of immune cells; HSD reduced subcutaneous tumors and lung metastases through TSC2-mediated metabolic shutdown, MITF-related redifferentiation, and cell-cycle arrest.

**Table 2:** Population studies and systematic-review evidence on sodium intake and cancer risk.

Reference	Model	Salt treatment	Cancer type	Direction of effect	Microbiota/immunity involved	Mechanistic summary
(41)	Prospective cohort of middle-aged Japanese adults	Estimated total salt intake and intake of high-salt foods	Gastric cancer	Increased risk	Population association	Gastric cancer risk increased with higher intake of salt and salt-preserved foods; associations with total salt and multiple salt-preserved foods were clearer in men.
(19)	Epidemiological review	Intake of salt and salt-preserved foods	Gastric cancer	Increased risk	Summary of population evidence	The review summarized evidence that salt-preserved foods may increase the risk of H. pylori infection and gastric cancer and may act synergistically with H. pylori to promote gastric cancer.
(24)	Meta-analysis of prospective studies	Habitual dietary salt intake	Gastric cancer	Increased risk/dose trend	Summary of population evidence	In prospective population studies, dietary salt intake was directly associated with gastric cancer risk, with risk increasing as intake increased.
(42)	Systematic review/meta-analysis of salt intake and gastric cancer	High vs low salt intake	Gastric cancer	Increased risk	Summary of population evidence	The systematic review showed that high salt intake, pickled foods, and processed meats were associated with increased gastric cancer risk and discussed mechanisms involving gastric mucosal injury and H. pylori colonization.
(43)	UK Biobank prospective cohort	Frequency of adding salt at the table; urinary sodium validation	Gastric cancer	Increased risk	Population association	Participants who “always” added salt to food had an approximately 41% higher risk of gastric cancer than those who never or rarely added salt, with a dose trend supported by urinary sodium estimates.
(44)	Systematic review/meta-analysis of observational studies on esophageal cancer	Dietary salt/sodium intake	Esophageal cancer	Increased risk/heterogeneous evidence	Summary of population evidence	The meta-analysis evaluated dietary salt intake and esophageal cancer risk; the findings suggested that high salt/sodium intake may be associated with increased esophageal cancer risk, although study type and region introduced heterogeneity.
(45)	Netherlands Cohort Study; 120,852 participants; 17.3 years of follow-up	Dietary sodium, potassium, and fluid intake	Renal cell carcinoma	Increased risk	Population association	Sodium intake was associated with increased RCC risk; risk was further elevated with high sodium plus low fluid intake, suggesting that salt-related cancer risk is not limited to gastric cancer.
(46)	Population study stratified by H. pylori infection, smoking, tumor site, and histology	Salt intake	Gastric cancer	Increased risk	Population association	The study supported salt intake as an important dietary risk factor for gastric cancer, and the risk differences were not fully explained by H. pylori infection, smoking, tumor site, or histological type.

## The role of immune responses in the relationship between HSD and cancer

### Immunoediting

Chronic inflammation is an established hallmark of cancer development (47). Recent studies suggest that HSD can affect the immune system by promoting inflammatory responses and may also participate in immune processes involved in tumor initiation and progression. In contrast to the previous section, which focused on immune activation and inflammatory responses, this section emphasizes immunoediting. Immunoediting includes three stages: immune elimination, immune equilibrium, and immune escape. In the early stage, an HSD-induced proinflammatory environment may enhance immune surveillance and help eliminate nascent tumor cells. However, under long-term or chronic exposure, persistent inflammation and immune pressure may lead to immune-cell exhaustion or skewing toward protumor phenotypes, allowing tumor cells to survive and expand through immune escape. This bidirectional effect suggests that the impact of HSD on the immune system is not limited to simple proinflammatory activation. Instead, HSD may dynamically alter the composition and function of the TME, thereby influencing cancer initiation and progression.

### Cytokines and T cells

In salt-sensitive rats, HSD has been shown to induce an increase in renal IL-6 (48). High salt also induces the production of macrophage inflammatory protein-2 (MIP-2) and TNF-α and synergistically enhances lipopolysaccharide- or TNF-α-induced MIP-2 expression (30). Animal studies indicate that high-salt environments increase the overall activation level of the immune system by promoting secretion of proinflammatory cytokines. *In vitro* experiments further show that increasing NaCl concentration by 40 mmol/L promotes IL-2 expression and T-cell proliferation, and induces polarization of naive CD4^+^ T cells toward an inflammatory Th17 phenotype through the SGK1- and NFAT5-mediated p38/MAPK pathway (14, 15, 49–51). Salt-induced CD4^+^ T-cell polarization depends on IL-23 signaling and is closely associated with activation of NFAT5 (also known as TonEBP) and SGK1. In addition, high salt suppresses anti-inflammatory activity (17). Studies show that increased NaCl not only impairs regulatory T-cell (Treg) function, but is also associated with increased IFN-γ secretion *in vitro* and *in vivo (*36, 52). Further evidence indicates that high salt can drive anti-inflammatory CD4^+^FoxP3^+^ Treg cells toward an inflammatory Th1 phenotype, accompanied by increased IFN-γ secretion (37, 53, 54). In addition to CD4^+^ T cells, NaCl has also been shown to activate CD4^+^ T cells and promote effector phenotype differentiation (4), while suppressing terminal differentiation of CD8^+^ T cells and enhancing their effector function (7). Overall, high salt shifts the immune system toward an inflammatory and activated state by promoting proinflammatory cytokine secretion, enhancing Th17 differentiation, weakening Treg function, and altering the effector state of CD8^+^ T cells. This imbalance in immune regulation not only aggravates chronic inflammation, but may also affect immunoediting and promote tumor cell immune escape, thereby driving cancer initiation and progression.

### Macrophages, NK cells, and dendritic cells

Two macrophage phenotypes are widely recognized: classically activated proinflammatory M1 macrophages induced by IFN-γ in T helper-type responses, and M2 macrophages induced by Th2 cytokines, such as IL-4 and IL-13, which suppress inflammation and promote tissue repair (40). HSD can promote macrophage transition toward a proinflammatory M1 phenotype by activating the p38/cFos or Erk1/2/cFos pathways. At the same time, it weakens anti-inflammatory M2 macrophage function by inhibiting glycolysis and mitochondrial metabolic output (41, 55, 56). This shift disrupts the M1/M2 balance and promotes a sustained proinflammatory state. Beyond macrophage regulation, high salt also has complex and context-dependent effects on NK cell function. Some studies have found that HSD can enhance the antitumor function of NK cells by reducing PD-1 expression and increasing IFN-γ and serum hippurate levels (10). However, other studies show that HSD reduces NK cell numbers in the spleen and lung of mice and inhibits NK cell maturation in the bone marrow. This process is associated with ROS signaling-mediated downregulation of CD122, which reduces NK cell responsiveness to IL-15 and impairs NK cell function (18). These differences suggest that the effects of high salt on NK cells may depend on disease context and experimental conditions. In addition, high salt can interact with DCs. Studies show that high salt enhances the formation of IsoLG protein adducts in mouse DCs and promotes IL-1β secretion, thereby enhancing T-cell activation and inducing IL-17A and IFN-γ secretion (43). These changes further promote Th17/Th1-type immune responses.

### Stage-dependent immune consequences of HSD exposure

Evidence from preclinical and human studies indicates that the immune system has dual and antagonistic roles in tumor initiation and progression, a process known as immunoediting. Immunoediting includes three stages: immune elimination, immune equilibrium, and immune escape. In the early stage, the immune system recognizes and eliminates abnormal cells through immune elimination. During equilibrium, the immune system coexists with residual tumor cells over long periods and applies strong selective pressure. In later stages, tumor cells evade immune clearance through immune escape mechanisms, thereby promoting tumor progression (44, 53, 57). High salt induces proinflammatory immune responses, can exacerbate autoimmune states, and under certain conditions may transiently enhance immune surveillance, thereby potentially promoting the elimination phase of immunoediting. During early tumorigenesis, NaCl may promote tumor cell clearance through immune surveillance. However, *in vitro* studies of CD4^+^ T cells show that prolonged high-salt exposure can lead to T-cell dysfunction and exhaustion (10). Similarly, long-term *in vitro* high-salt treatment of human monocytes can induce polarization toward an M2 macrophage phenotype, which has immunosuppressive and protumor properties (47). These data indicate that the effects of NaCl on the immune system are stage-dependent: during early tumor immunoediting, NaCl may enhance elimination, whereas at later stages it may increase tumor immunogenicity by promoting immune exhaustion and immune escape. Together, these findings suggest that HSD reshapes antitumor immunity through coordinated effects on immunoediting, cytokine signaling, T-cell differentiation, macrophage polarization, NK-cell activity, and DC-mediated T-cell activation, with outcomes that depend on exposure duration and tumor stage (**Figure 3**).

**Figure 3:**
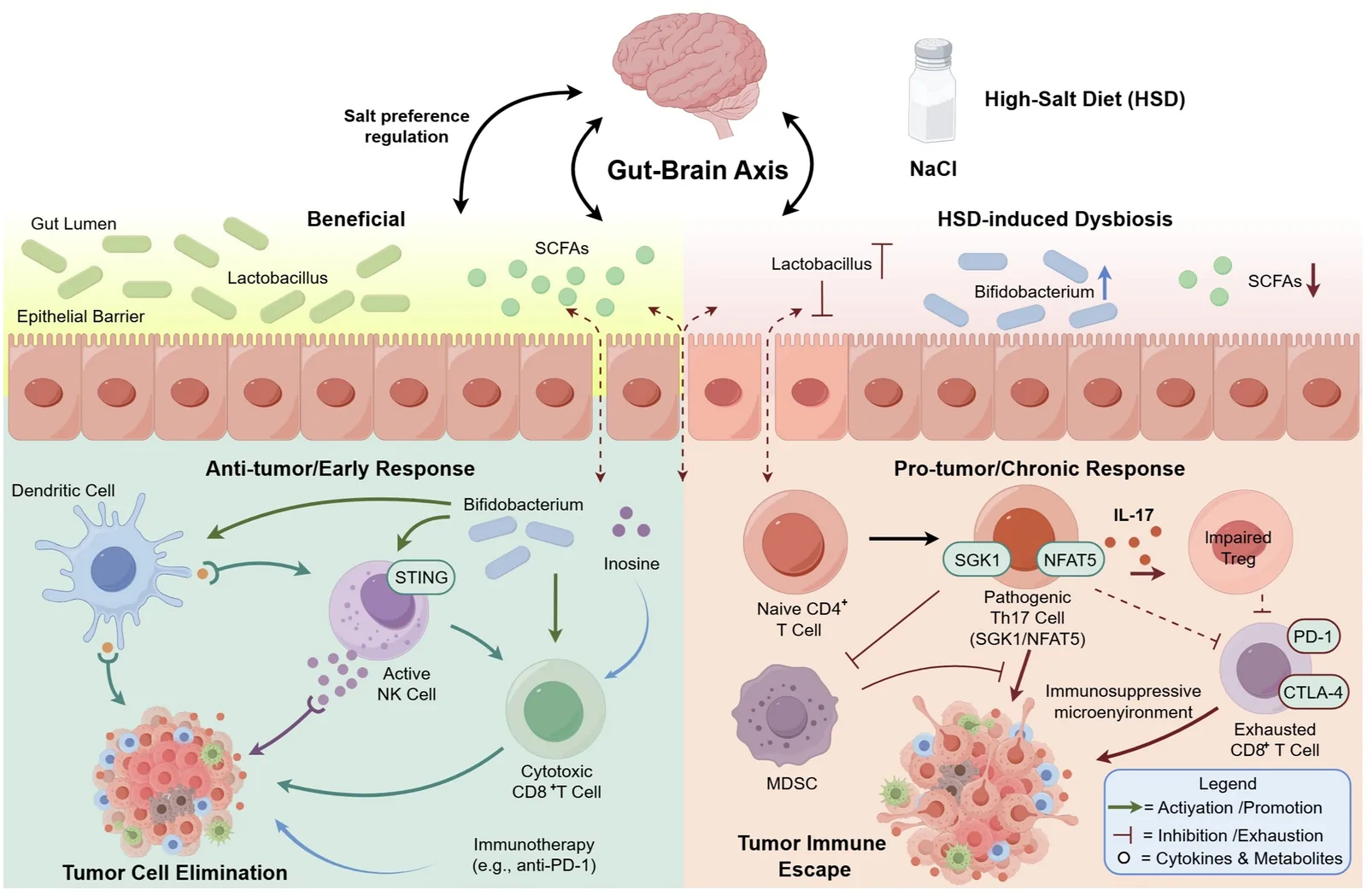
Metabolite-mediated immune regulation driven by the gut microbiota. This image summarizes how microbial metabolites, including SCFAs, BAs, and Trp metabolites, connect gut microbiota changes with immune regulation. These metabolites act through receptors and pathways such as FFAR2/FFAR3, TGR5/FXR/ROR γ t, and AhR to regulate macrophages, DCs, Treg cells, Th17 cells, and inflammatory tolerance.

## Microbiota-dependent immune regulation by microbial metabolites

Dynamic interactions occur between the gut microbiota and the host immune system, including both innate and adaptive immunity, and these interactions are essential for maintaining intestinal homeostasis and suppressing inflammation (54, 58). Intestinal epithelial cells and immune cells, such as DCs, macrophages, and regulatory T cells (Treg cells), serve as bridges between the gut microbiota and the immune system. These cells can sense and respond to the intestinal microenvironment, helping to maintain immune tolerance, activate specific immune responses, and resist invasion by exogenous pathogens. Metabolites produced by the gut microbiota, mainly SCFAs and BAs, bind to receptors on the surface of immune cells, alter cellular signal-transduction pathways, and regulate immune-cell gene expression, thereby influencing immune-cell differentiation and function. These metabolites play key roles in regulating immune-cell differentiation and function, particularly in maintaining immune tolerance and suppressing excessive immune responses. These effects are crucial for maintaining both intestinal and systemic immune homeostasis (59).

### SCFAs in immune regulation

Expression of the GPR43 gene, also known as FFAR2, is closely associated with several important innate immune receptors, including Toll-like receptors (TLR2 and TLR4), formyl peptide receptors (FPR1 and FPR2), IL8RB, also known as CXCR2, and C5aR (56). Studies have shown that SCFAs, especially acetate and propionate, regulate dendritic cell (DC) function through the G protein-coupled receptors GPR43 (also known as FFAR2) and FFAR3, thereby promoting the resolution of intestinal inflammation (56, 60). SCFAs can also promote SCFA absorption by activating proton-coupled monocarboxylate transporter 1 (MCT1) and sodium-coupled monocarboxylate transporter 1 (SMCT1) (61). In addition, SCFAs can activate a series of G protein-coupled receptors (GPCRs), including FFAR2 (GPR43), FFAR3 (GPR41), hydroxycarboxylic acid receptor 2 (HCAR2, GPR109a), and Olfr-78 (OR51E2), and activation of these receptors has important roles in immune responses (60, 62).

Macrophages are generally divided into two types: inflammatory macrophages (iMacs) and immune-tolerant macrophages (tMacs). iMacs participate in inflammatory immune responses, whereas tMacs suppress inflammation and maintain homeostasis by producing large amounts of IL-10 and TGF-β (59). This indicates that macrophages can exert both pro-inflammatory and anti-inflammatory effects during immune responses. SCFAs can regulate the polarization state of macrophages, inhibit activation of M1-type, pro-inflammatory macrophages, and promote differentiation of M2-type, anti-inflammatory macrophages, which is important for controlling local and systemic inflammatory responses. Experimental studies have shown that SCFAs can significantly reduce histone deacetylase (HDAC) mRNA expression in monocytes and macrophages, decrease production of inflammatory factors, and contribute to control of systemic inflammation (63, 64). After activation, macrophages produce large amounts of inflammatory mediators, including TNF-α, IL-1β, IL-6, chemokines, and nitric oxide (NO). Among SCFAs, butyrate can negatively regulate NLRP3 (NOD-like receptor thermal protein domain-associated protein 3)-mediated inflammatory signaling and suppress macrophage activation (64, 65). In addition, butyrate promotes an anti-inflammatory tolerant phenotype in macrophages by reprogramming macrophage metabolism toward oxidative phosphorylation. This not only restores the hyporesponsive features of intestinal macrophages but also prevents T-cell dysfunction, thereby helping to maintain intestinal immune tolerance (64, 66).

SCFAs can promote the maturation of DCs and regulate immune responses through immune tolerance by modulating their differentiation toward specific T-cell subsets, such as Treg cells. SCFAs act directly on T cells through FFAR2 and FFAR3 receptors to regulate their differentiation and function, and they play an especially important role in Treg-cell differentiation. Butyrate is a potent inducer of Treg-cell differentiation. By inhibiting histone deacetylase (HDAC) activity, it promotes Foxp3 gene expression and thereby enhances the immunosuppressive effects of Treg cells (59, 64). This mechanism helps maintain intestinal immune tolerance and prevents intestinal inflammation and autoimmune responses. By promoting Treg-cell differentiation and suppressing the release of inflammatory factors, SCFAs help maintain intestinal immune homeostasis. Through regulation of DCs, SCFAs, especially butyrate, can also suppress colonic inflammatory responses and reduce tumor-promoting effects. The immunomodulatory effects of SCFAs are essential for intestinal immune tolerance, helping to maintain the stability of the intestinal immune system so that it can effectively resist pathogen invasion while avoiding excessive responses to resident microorganisms and food antigens.

### Effects of BAs on immune cells

BAs are important signaling molecules produced by cholesterol metabolism and exert regulatory effects in the immune system. They act on immune cells by activating multiple receptors, including G protein-coupled bile acid receptor 1 (GPABR1 or TGR5), farnesoid X receptor (FXR), and the more recently identified retinoic acid receptor-related orphan receptorγ t (ROR γ t) (67–69). BAs regulate tolerogenic responses in the immune system by activating FXR and TGR5 and antagonizing ROR γ t. Through these receptor-mediated effects, BAs promote macrophage polarization toward the anti-inflammatory M2 phenotype and promote T-cell differentiation toward immune-tolerant Treg cells, thereby upregulating IL-10 production and suppressing inflammatory responses. By reducing the production of pro-inflammatory cytokines, such as IL-6, IL-1β, TNF-α, and IL-17, BAs inhibit activation of DCs, ILC3, and Th17 cells, thereby modulating immune responses (69–74).

Both TGR5 and FXR can be expressed in DCs isolated from the intestine and liver. TGR5 activation promotes macrophage polarization toward a tolerogenic M2 phenotype (70). TGR5 activation not only promotes M2 polarization of macrophages but also exerts anti-inflammatory effects by inhibiting LPS-induced cytokine release and blocking NLRP3 inflammasome-dependent inflammatory responses (75–77). FXR is also essential for maintaining the tolerogenic phenotype of macrophages. Through SHP-dependent and SHP-independent anti-inflammatory mechanisms, FXR regulates the expression of multiple genes involved in inflammatory responses (69). DCs are the most effective antigen-presenting cells and can activate naive T cells and regulate immune responses (54, 59). BAs regulate DC maturation and antigen-presentation function by binding to the TGR5 receptor. Studies have found that TGR5 activation can induce human monocytes to differentiate into DCs that produce low levels of IL-12 and TNF-α (78). The secondary bile acid derivative isoDCA confers an anti-inflammatory phenotype on DCs by limiting FXR activity in DCs. This effect is essential for intestinal immune tolerance and helps reduce excessive immune and inflammatory responses (78).

BAs control host immune responses by directly regulating the balance between Th17-cell and Treg-cell differentiation. The BA derivatives isoalloLCA and 3-oxoLCA promote the production of mitochondrial reactive oxygen species (mitoROS). Treg-cell differentiation depends mainly on oxidative phosphorylation (oxPhos) after exposure to BA derivatives. Therefore, increased mitoROS production can promote Treg-cell differentiation. Th17-cell differentiation can be blocked by inhibiting ROR γ t function (59). 3-oxoLCA and isoLCA directly bind to ROR γ t and inhibit its function, thereby suppressing Th17-cell differentiation (71–73). ROR γ t is expressed mainly in Th17 cells and group 3 innate lymphoid cells (ILC3). It is essential for the differentiation of these cells during chronic inflammation and autoimmune disease (72, 74). Indeed, inhibition of ROR γ t not only reduces the frequency of Th17 cells but also helps maintain immune tolerance and suppress excessive immune responses, particularly in intestinal inflammation.

### Other metabolites

Trp metabolites include products generated by direct microbial conversion of Trp in the gut, such as indole, tryptamine, indole ethanol (IE), and indole propionic acid (IPA), as well as metabolites produced through the kynurenine (Kyn) pathway, such as 3-HAA and 3H-Kyn (62, 79, 80). Trp metabolites play important roles in the differentiation and function of Treg cells, Bregs, ILC3, and anti-inflammatory macrophages (80). *In vitro* studies show that Trp metabolites suppress inflammatory responses in macrophages through AhR signaling, reduce histamine production, and downregulate secretion of the proinflammatory cytokine IL-6 (81). The downstream Kyn metabolite 3-HAA reduces IL-6 and TNF-α production in macrophages by inhibiting the PI3K/Akt/mTOR and NF-kappaB signaling pathways (64). Thus, metabolites derived from microbial Trp conversion and the Kyn pathway can suppress macrophage inflammatory responses and contribute to immune tolerance (**Figure 3**).

## Immune regulation of gut microbiota homeostasis

The immune system is one of the core host factors shaping both normal and dysbiotic gut microbiota composition through mucosal barrier function, immune molecules such as IgA and antimicrobial peptides, and immune-cell responses. Therefore, clarifying the crosstalk between host immunity and the microbiome is important for understanding how immune changes directly or indirectly drive gut microbiota dysbiosis (58, 82). Gut-associated lymphoid tissue (GALT) contains large numbers of immune cells and is considered one of the largest immune organs in the body. Recent studies have shown that changes in innate immunity, including complement and NOD-like receptor signaling, and adaptive immunity, including the Treg/Th17 balance and IgA secretion, can markedly affect gut microbiota composition, function, and diversity.

### Innate immunity

The innate immune system senses microbial components through pattern-recognition receptors (PRRs) and plays a key role in regulating gut microbiota composition and homeostasis. PRR-mediated microbial sensing influences microbial colonization in the mouse intestine (82). Loss of MYD88 signaling in epithelial cells increases mucosa-associated bacteria, promotes bacterial translocation to mesenteric lymph nodes, and markedly alters microbiota composition (83). NOD-like receptors (NLRs) are also closely associated with dysbiosis. NOD1 normally maintains microbiota homeostasis by recognizing peptidoglycan from Gram-negative bacteria. NOD1-deficient mice show expansion of bacterial populations, including commensal Clostridia, Bacteroides, segmented filamentous bacteria (SFB), and Enterobacteriaceae (84). Similarly, Nod2-/- mice exhibit pronounced microbiota imbalance, which increases susceptibility to intestinal inflammation and colorectal cancer (85–87). These findings indicate that defects in innate immune sensing can directly cause dysbiosis and increase host susceptibility to disease. In addition, NLRP6, an intestinal epithelial inflammasome-related protein, has an important role in maintaining gut microbiota stability. Metabolites derived from commensal microorganisms can activate the NLRP6 inflammasome and regulate microbiota composition through feedback mechanisms (88–90). Earlier studies also showed that loss of inflammasome components, such as ASC and caspase-1, causes dysbiosis (72), further supporting the central role of inflammasomes in microbiota homeostasis.

### Adaptive immunity

Adaptive immunity also has an important role in regulating gut microbiota composition and homeostasis. B cell-mediated microbiota control through secretory IgA (sIgA) is a core mechanism by which adaptive immunity maintains intestinal homeostasis. sIgA can precisely target specific bacterial species and even phenotypic features of specific strains (91, 92). When serum lipopolysaccharide (LPS) levels exceed the regulatory capacity of sIgA, gut microbiota composition undergoes moderate changes (92). In mice deficient in activation-induced cytidine deaminase (AID), impaired somatic hypermutation reduces sIgA production, thereby promoting increased colonization by segmented filamentous bacteria and expansion of anaerobic bacteria. Restoration of IgA can reverse these abnormalities (93–95). In addition, follicular helper T cells (TFH), a specialized subset of CD4^+^ T cells, promote B-cell IgA selection and thereby participate in microbiota control. TFH cells highly express the inhibitory receptor PD-1, and this pathway contributes to microbiota regulation and IgA production. PD-1-deficient mice show a reduced proportion of IgA-coated bacteria, accompanied by reduced Bifidobacterium and expansion of Enterobacteriaceae (96).

### Immune overactivation

Excessive activation of the immune system, especially in autoimmune diseases and chronic inflammatory conditions such as Crohn’s disease, ulcerative colitis, and rheumatoid arthritis, can markedly alter gut microbiota composition and diversity (97, 98). Immune activation is often accompanied by enhanced inflammation. Inflammation disrupts the intestinal barrier and affects microbial colonization by changing local nutrient availability, redox status, and mucosal niches. In patients with autoimmune diseases, abnormal immune activation is closely associated with dysbiosis. For example, the microbiota of patients with Crohn’s disease and ulcerative colitis is often characterized by reduced diversity and enrichment of pathogenic bacteria, including pathogenic Escherichia coli, Clostridioides difficile, and Fusobacterium nucleatum (97–99). This dysbiosis can further aggravate intestinal inflammation, forming a vicious cycle between inflammation and microbial imbalance. Immune overactivation is also often accompanied by excessive release of proinflammatory cytokines, such as TNF-α, IL-1β, and IL-6. Barrier disruption allows bacteria and their metabolites, including endotoxin, to enter the bloodstream more readily, thereby inducing systemic inflammatory responses.

### Immune-microbiota balance and dysbiosis

Overall, the immune system has a central role in maintaining gut microbiota homeostasis. Innate immunity senses and regulates microbial components through PRRs, including Toll-like receptors, NOD-like receptors, and inflammasomes. Defects in these pathways often lead to microbiota imbalance and increase host susceptibility to inflammation and tumorigenesis. Adaptive immunity mainly relies on coordinated actions of B cell-derived IgA and T cells, such as TFH cells, to precisely regulate specific bacterial communities or strain phenotypes, thereby maintaining microbiota diversity and stability. However, when the immune system becomes overactivated, especially in autoimmune diseases and chronic inflammatory states, microbiota homeostasis is disrupted, with reduced diversity and enrichment of pathogenic bacteria. This immune imbalance interacts with dysbiosis to form a vicious cycle of amplified inflammation. Ultimately, this process not only damages the local intestinal environment but may also trigger systemic inflammatory responses through barrier disruption and proinflammatory-factor release. Together, these findings indicate that the dynamic balance of immune-microbiota interactions is essential for intestinal health. They also provide a basis for understanding how immune disruption may reshape the gut microbiota and contribute to inflammation-associated tumorigenesis (**Figure 4**).

**Figure 4:**
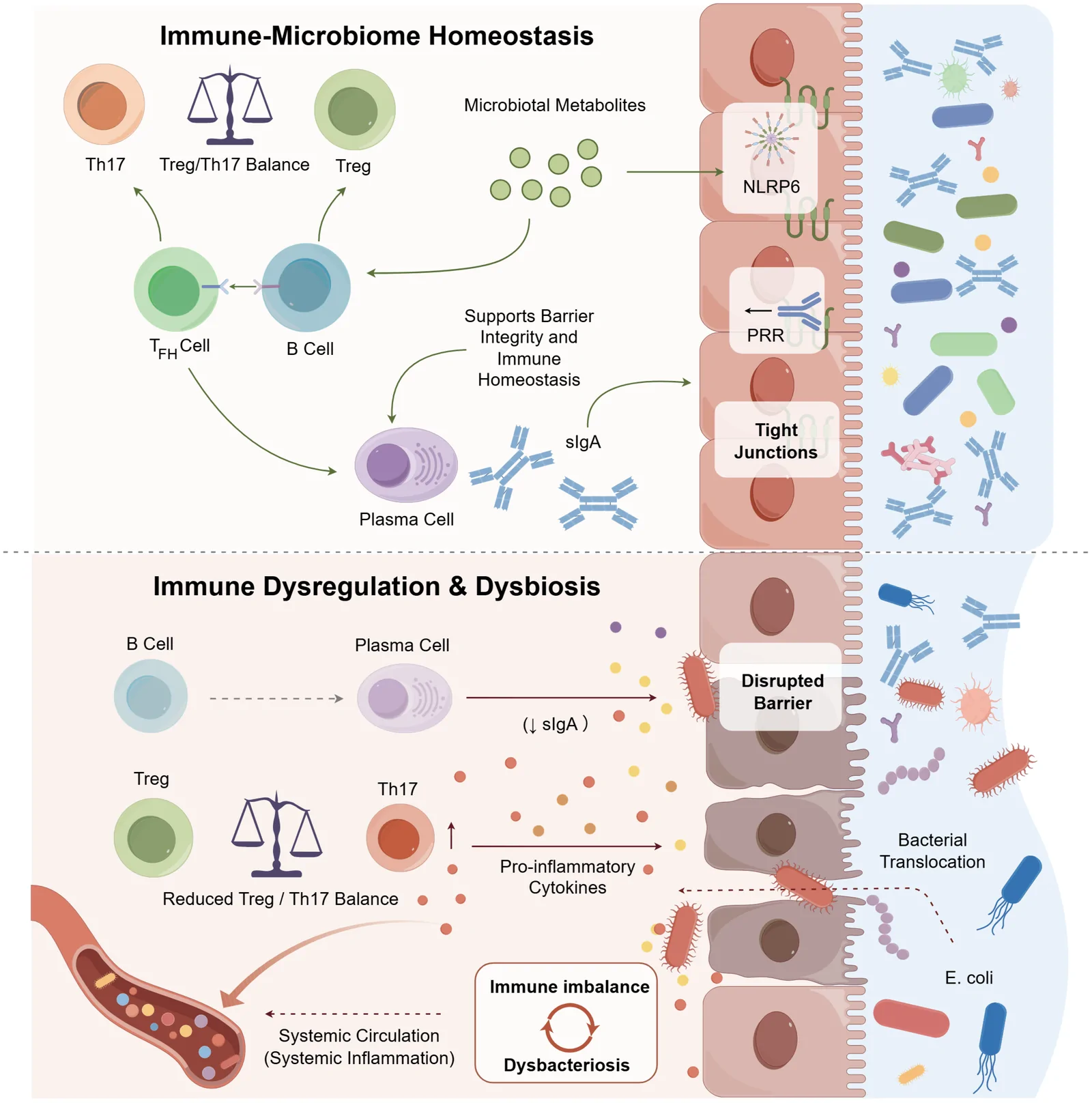
Immune-gut microbiota interactions from immune change to dysbiosis. This image emphasizes the reverse direction of the microbiota-immune axis, showing how innate and adaptive immune mechanisms shape microbial composition. Barrier function, PRRs, inflammasomes, IgA, and T-cell responses help maintain microbial homeostasis, whereas excessive immune activation can drive dysbiosis and reinforce inflammation.

## Microbiota-dependent effects of HSD on immune regulation

In recent years, the gut microbiota has been shown to occupy an intermediate position in host physiological responses induced by HSD. HSD affects the host by modulating the gut microbiota, particularly the abundance of specific microbial groups and the production of microbial metabolites (4, 100–104). Researchers have found that HSD can markedly reshape the gut microbiota: the Firmicutes/Bacteroidetes ratio increases, the abundances of Lachnospiraceae and Ruminococcus increase, whereas the abundance of Lactobacillus decreases substantially (4). A high NaCl content in the diet alters microbial composition and function, reduces the relative abundance of Lactobacillus, and promotes a pro-inflammatory state in the intestine.

In studies examining the effects of HSD on microbial communities, analyses at the genus and operational taxonomic unit (OTU) levels identified four bacterial genera that were significantly associated with HSD. Among them, Lactobacillus showed the strongest association: its relative abundance decreased by approximately twofold after 4 weeks of HSD exposure and rapidly recovered after return to a control diet (r = −0.703, q = 0.003). The relative abundance of an unclassified genus within Clostridiales decreased by more than onefold after 4 weeks of HSD and partially rebounded after 1 week on the control diet (r = −0.605, q = 0.017). By contrast, Lachnospiraceae and Oscillospira were positively associated with HSD (r = 0.614, q = 0.017 and r = 0.548, q = 0.042, respectively). In addition, three fecal OTUs were significantly associated with the duration of HSD exposure: Lactobacillus (r = −0.707, q = 0.005), OTU#9 (Clostridiales, r = −0.684, q = 0.005), and OTU#25 (Lachnospiraceae, r = 0.609, q = 0.027) (4).

Further experimental studies showed that, compared with low-salt (0.03% NaCl) or control (0.5% NaCl) diets, high-salt diets (4% and 10% NaCl) significantly altered the composition and functional characteristics of the microbiota: high salt depleted lactic acid-producing bacteria in a dose-dependent manner, although these bacteria have important protective roles in salt-sensitive inflammatory diseases (103). Consistent with the study by Wilck et al., HSD primarily altered microbial structure by reducing the level of Lactobacillus murinus and was associated with pro-inflammatory immune responses. Decreased abundances of Lactobacillus, Oscillospira, Pseudoflavonifractor, and Clostridium were also observed (86), and supplementation with Lactobacillus murinus reversed this pro-inflammatory effect.

In addition, experimental studies have found that HSD increases the abundance of Bifidobacterium, enhances gastric colonization by H. pylori, and raises the abundance of relatively salt-tolerant species such as Escherichia coli (8, 21, 24). Results from 16S rRNA metagenomic analysis also showed that, compared with healthy controls, HSD-fed mice exhibited increased overall microbial richness and evenness. These changes not only reflect the effects of HSD on the gut microecology, but are also closely related to intestinal barrier injury, immune system alterations, and the production of microbial metabolites.

Gut microbial metabolomics has further revealed the molecular-level effects of HSD on the microbiota. Different microbial communities metabolize xenobiotics and dietary components in distinct ways, thereby producing different effects on host gene expression and substance metabolism. Changes in metabolites not only shape the metabolomic features of the intestine, but may also have profound effects on host physiological functions, while changes in host phenotype can in turn regulate microbial communities (104). For example, HSD may influence the metabolic potential of the microbiota and the generation of metabolic products by regulating gene expression in the gut microbiota, particularly genes related to salt tolerance (4). These findings suggest that HSD not only remodels microbial structure at the macroscopic level, but also changes the functional properties of the microbiota at the molecular level, providing a basis for subsequent studies of its effects on host immunity and disease susceptibility.

### HSD-induced changes in microbiota-derived metabolites

Beyond changes in microbial composition, HSD also alters microbial metabolic output. Because microbiota-derived metabolites are key mediators of host immune regulation, HSD-induced changes in SCFAs, BAs, and Trp metabolites may provide an important mechanistic link between dietary salt, dysbiosis, immune disturbance, and disease susceptibility.

### Reduced SCFA production under HSD

Current evidence indicates that HSD significantly reduces SCFA production, particularly butyrate. Analyses of gut microbiota dynamics and SCFA levels showed that HSD altered fecal microbial metabolic function and reduced the relative abundance of major SCFA-producing bacteria, including Lachnospiraceae, Clostridiaceae, and Oscillospira. Among seven measured SCFAs, butyrate was the most clearly affected, showing a marked decrease after 4 weeks of HSD exposure (4, 102). Because butyrate is important for intestinal barrier integrity and immune homeostasis, reduced butyrate production may be a key pathway through which HSD promotes intestinal inflammation and immune imbalance.

Some butyrate-producing bacteria can use lactate to synthesize high concentrations of butyrate (105). Therefore, the marked reduction in Lactobacillus under HSD conditions may indirectly reduce butyrate levels. This suggests that HSD disrupts the microbial metabolic network by affecting key butyrate-producing bacteria and their substrate supply, thereby leading to insufficient SCFA generation.

### Altered BA metabolism under HSD

BAs are synthesized in the liver and stored in the gallbladder, and they mainly participate in fat digestion and absorption through the intestine. The gut microbiota can transform primary BAs through reactions such as dehydroxylation and deamination, generating various secondary metabolites and playing a key role in BA metabolism.

In the intestine, Clostridium cluster XIVa (including Clostridium scindens, C. hiranonis, and C. hylemonae) and cluster XI (C. sordellii) can catalyze 7α/β-dehydroxylation reactions through enzymes encoded by the BA-inducible (bai) operon, converting primary 7α-hydroxy BAs (such as cholic acid, CA, and chenodeoxycholic acid, CDCA) into the secondary BAs deoxycholic acid (DCA) and lithocholic acid (LCA), respectively (106).

HSD may affect BA metabolism by altering the relative abundance of Clostridium in the intestine. Studies have shown that Bacteroides can dehydroxylate primary BAs to generate secondary BAs (107), and when HSD inhibits this activity, the levels of secondary BAs decrease. At the same time, salt-tolerant bacteria, such as Escherichia coli, may enhance BA metabolism under high-salt conditions, potentially increasing the risk of intestinal inflammation and further affecting lipid and cholesterol metabolism through the gut-liver axis.

### Disrupted Trp metabolism under HSD

Trp is an essential amino acid that is mainly metabolized by spore-forming Clostridium, Ruminococcus, and Lactobacillus within the intestinal Firmicutes (80, 108, 109). Trp can be converted through multiple metabolic pathways into various downstream products, including indole derivatives, kynurenine, and kynurenine derivatives.

Studies have shown that indolepropionic acid (IPA) can inhibit the early development of breast cancer by acting on AHR and PXR (110). IPA is mainly produced through microbial metabolism by Bacteroides, Clostridium, Lactobacillus, Parabacteroides, Peptostreptococcus, and other microorganisms (108). In contrast, kynurenine is a ligand of the aryl hydrocarbon receptor (AhR), which can promote cell migration and immune tolerance and exert pro-tumor effects during cancer progression (111). During Trp metabolism, indoleamine 2,3-dioxygenase 1 (IDO1) is the rate-limiting enzyme for the conversion of Trp to kynurenine. Gut microbiota members, such as Lactobacillus, Pseudomonas aeruginosa, Pseudomonas, and fluorescent bacteria (103, 109), can increase kynurenine production by promoting IDO1 expression, thereby driving cancer cell metastasis under certain conditions.

HSD can indirectly regulate Trp metabolite generation by altering the relative abundance of Firmicutes and Lactobacillus (4, 103, 109). These changes suggest that a high-salt environment may disrupt the balance of Trp metabolism, thereby changing the composition and biological effects of downstream metabolites (**Figure 5**).

**Figure 5:**
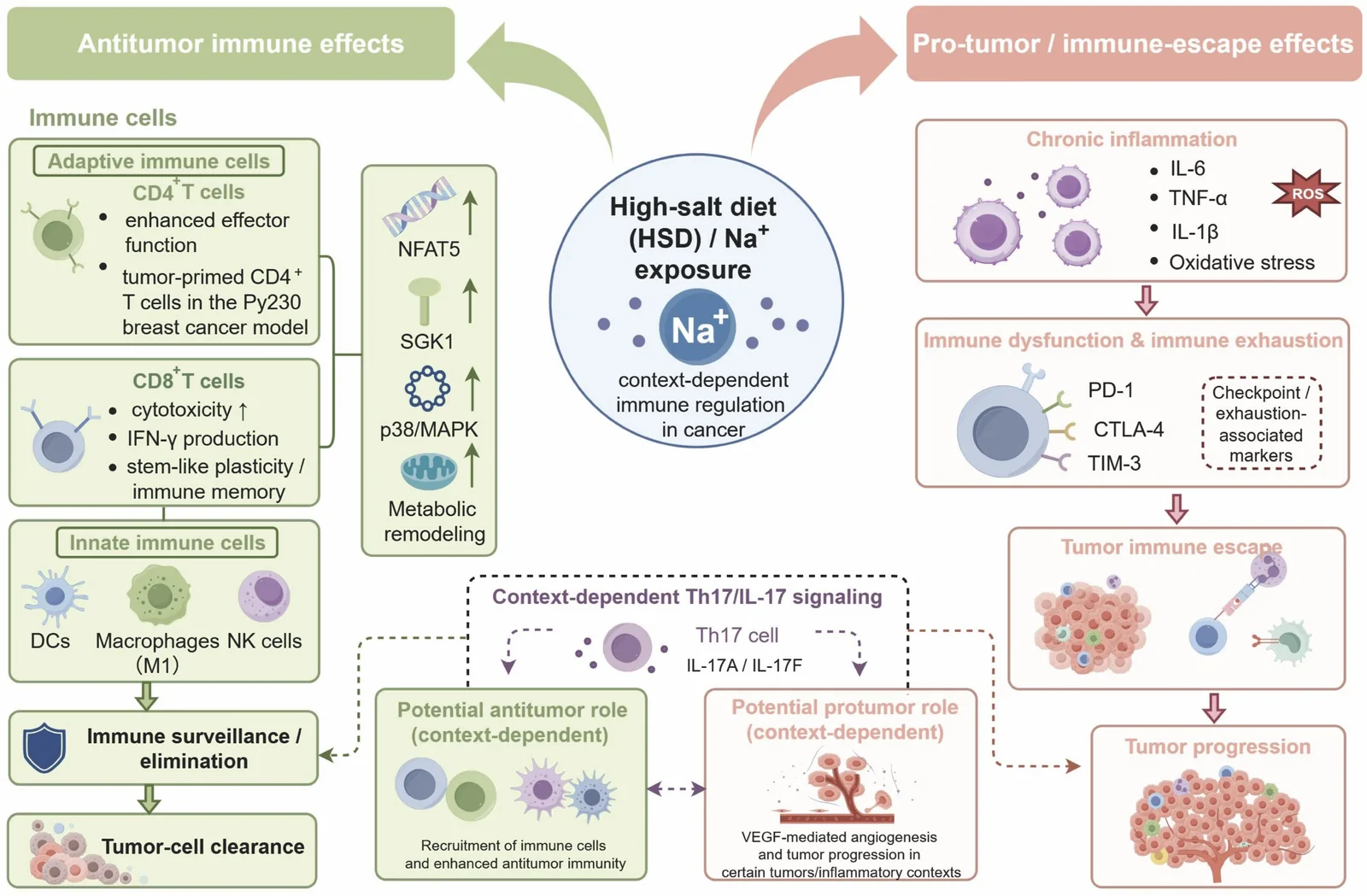
Effects of HSD on the structure and function of the gut microbiota. This image summarizes how HSD reshapes gut microbial ecology and metabolic output. The main changes include altered bacterial abundance, reduced Lactobacillus, disturbed SCFA production, modified BA and Trp metabolism, and downstream effects on barrier integrity and inflammatory signaling.

## Reverse regulation of dietary salt intake by the gut microbiota

Dietary salt intake not only directly affects host health but also alters the composition and function of the gut microbiota. Notably, recent studies suggest that the gut microbiota may, in turn, influence host preference for salt intake through several potential mechanisms.

Existing evidence indicates that the microbiota-gut-brain axis may have an important role in this process. Proposed mechanisms include changes in autonomic nervous and sensorimotor pathways (112, 113), immune activation such as inflammatory responses (113), and regulation of neuroendocrine pathways (114). Among these pathways, gut-derived hormones, including GLP-1, PYY, and ghrelin, can act on vagal afferent fibers and hypothalamic feeding centers, thereby influencing neural circuits related to salt taste and salt appetite (115).

Animal studies provide preliminary support for this concept. In both salt-sensitive and salt-tolerant rats fed an HSD, the proportion of Firmicutes was increased (116), suggesting that this phylum may be associated with salt preference or salt tolerance. Other studies suggest that high salt can promote Th17-related immune responses and activate pathways related to osmotic pressure and ion homeostasis. These changes are accompanied by reductions in certain Lactobacillus species, suggesting that a microbiota-immune-neural pathway may link gut microbial changes with salt preference (4, 117). In addition, SCFAs produced by the gut microbiota can act on the central nervous system through immune and vagal signaling pathways, thereby affecting appetite and salt demand (118). SCFAs can also stimulate intestinal endocrine L cells through FFAR2/FFAR3 to release GLP-1 and PYY, which may indirectly regulate salt-related feeding behavior (119, 120). Other potential pathways include the effects of microbial Trp metabolites on intestinal mucosal inflammation and barrier function through AhR, as well as the involvement of microbially modified BAs in energy- and taste-related pathways through TGR5/FXR signaling (107, 121).

However, human evidence supporting microbiota-mediated reverse regulation of salt intake remains limited. Current findings are also affected by confounding factors, including interindividual differences, baseline salt sensitivity, dietary patterns, and medication use. Future longitudinal intervention studies and mechanistic experiments are needed to verify causality and identify the key microorganisms and metabolites involved. Overall, the gut microbiota may participate in salt taste perception and dietary choice through metabolite-mediated and neuroimmune pathways, and the microbiota-gut-brain axis provides a useful theoretical framework for understanding its potential regulatory role in salt intake behavior (118).

It should be emphasized that the proposed microbiota-HSD-gut-brain axis remains a hypothesis-generating framework rather than an experimentally established mechanism. Although existing studies separately support links between HSD and gut microbiota changes, between microbial metabolites and gut-brain communication, and between neuroimmune signaling and dietary behavior, direct experimental evidence integrating HSD, the gut microbiota, gut-brain signaling, and salt-intake preference is still lacking. Future longitudinal intervention studies, microbiota-transfer experiments, metabolomic analyses, and neuroimmune mechanistic studies are needed to clarify whether this proposed axis represents a causal regulatory pathway.

## Microbiota-dependent mechanisms in HSD-related cancer development

### Dysbiosis, barrier disruption, and cancer progression

Colorectal cancer and gastric cancer are among the most common malignancies of the digestive tract. Growing evidence indicates that the gut microbiota has a central role in tumorigenesis and immune regulation. Dysbiosis can promote cancer development through inflammatory responses and can also influence cancer progression and immunotherapy responses through microbial metabolites and immune pathways.

Alterations in gut microbiota composition can disrupt the mucosal barrier, trigger the release of inflammatory cytokines, including IL-1, IL-6, and IL-23, activate the Th17/IL-17 axis, and further drive STAT3 and NF-kappaB signaling. These events promote epithelial cell proliferation and malignant transformation (122–125). Under HSD conditions, dysbiosis may be more likely to induce chronic inflammation and increase the risk of DNA damage and mutation.

In gastric cancer, HSD can enhance expression of the H. pylori virulence gene cagA, leading to reduced gastric acid secretion and aggravated inflammation, thereby accelerating gastric carcinogenesis (21). Microbiota-derived metabolites also have dual roles in carcinogenesis. SCFAs can inhibit abnormal epithelial cell proliferation, promote apoptosis, and enhance CD8^+^ T-cell cytotoxicity (126–128). In contrast, some secondary BAs can induce Treg expansion and promote immune escape and tumor metastasis (129). HSD-induced dysbiosis is often accompanied by reduced butyrate levels and metabolic imbalance, which may represent an important mechanism by which HSD promotes carcinogenesis (102).

### The HSD-microbiota-immune axis in cancer regulation

The effects of HSD on tumor immunity depend partly on the gut microbiota. Studies have shown that Lactobacillus depletion weakens antitumor immunity mediated by DC maturation and Trp metabolites, thereby reducing Th17-related immune regulation and antitumor responses (4, 109). In contrast, some studies suggest that HSD may increase the abundance of Bifidobacterium. Colonization by Bifidobacterium in the TME can enhance NK-cell and CD8^+^ T-cell function and improve the efficacy of anti-CD47 monoclonal antibodies and immune checkpoint inhibitors (ICIs) by activating the STING pathway (130). Thus, HSD-microbiota-immune interactions are bidirectional. They may weaken antitumor immunity in some contexts, but may also enhance immunotherapy efficacy under specific conditions.

The gut microbiota influences cancer initiation and progression through multiple pathways, including inflammatory responses, barrier integrity, microbial metabolites, and immune-cell regulation. By altering microbial composition and metabolic output, HSD may exert complex bidirectional effects. On the one hand, it may promote inflammation and metabolic imbalance, thereby increasing carcinogenic risk. On the other hand, it may enhance antitumor immunity and ICI efficacy through specific bacterial taxa. These findings highlight the potential value of microbiota-diet-immunity interactions in cancer prevention and treatment.

### Gut microbiota changes in cancer therapy

Gut microbiota-derived metabolites have a substantial influence on the efficacy of tumor immunotherapy. SCFAs can enhance CD8^+^ T-cell-mediated antitumor immune responses, thereby improving tumor immunotherapy efficacy *in vitro* and *in vivo* (126–128). However, under HSD conditions, reduced butyrate levels may weaken immune responses and reduce therapeutic efficacy.

HSD may also improve therapeutic responses by increasing the abundance of Bifidobacterium pseudolongum. Its metabolite inosine can promote Th1-cell differentiation and antitumor immunity. In glucose-deprived environments, inosine can also serve as an alternative carbon source for CD8^+^ T cells, supporting their proliferation and effector function (131, 132). This process involves multiple mechanisms, including immunometabolic regulation, such as mTOR inhibition and AMPK activation, and epigenetic regulation, including histone acetylation and DNA methylation.

In addition, HSD-enhanced Bifidobacterium colonization may improve NK-cell function and promote tumor regression. When combined with anti-CD47 monoclonal antibodies, Bifidobacterium can enhance DC cross-presentation through STING signaling, thereby further activating CD8^+^ T-cell responses (130).

Interestingly, some studies have found that HSD can suppress enterotoxigenic Bacteroides fragilis (ETBF)-associated colorectal carcinogenesis by downregulating IL-17A and iNOS expression, suggesting a degree of protective activity (37).

Taken together, the gut microbiota has an important role in cancer therapy and immunotherapy. By altering microbiota composition and microbial metabolites, HSD may either weaken immunotherapy efficacy or improve immunotherapy responses under specific conditions. This provides a rationale for combining personalized dietary intervention with tumor immunotherapy.

## Potential intervention strategies targeting the gut microbiota

### Dietary intervention

The World Health Organization reports that the global average daily salt intake is approximately 10.8 g, more than twice the recommended level of less than 5 g/day. Reducing salt intake has therefore become an important strategy for promoting healthy diets (133). Epidemiological and experimental studies indicate that HSD can enhance the carcinogenic effects of H. pylori infection, particularly by promoting the expression of cagA-positive strains and increasing gastric cancer risk. Conversely, salt reduction may help lower the risk of H. pylori-associated gastric carcinogenesis (23).

Human and animal studies show that HSD can rapidly deplete Lactobacillus and is associated with increased Th17-cell responses and inflammation. Restoration of a normal diet or supplementation with Lactobacillus species, such as Lactobacillus murinus and Lactobacillus reuteri, can reverse these effects and reduce Th17 activation and inflammation (4, 134). These findings suggest that salt reduction may help maintain Lactobacillus balance, restore intestinal barrier function, and alleviate HSD-induced immune dysregulation.

However, the preventive value of low-salt diets in cancer remains to be defined. Although salt reduction is broadly feasible as a lifestyle intervention, its effects may vary according to baseline microbiota composition, dietary habits, salt sensitivity, and long-term adherence. Randomized controlled trials are needed to determine its practical role in cancer prevention and to identify populations most likely to benefit.

### Probiotics/prebiotics

The World Health Organization defines probiotics as “live microorganisms that, when administered in adequate amounts, confer a health benefit on the host”; Lactobacillus and Bifidobacterium are among the most extensively studied representatives and are widely regarded as generally recognized as safe (GRAS) (134, 135).

Probiotics can exert beneficial effects through multiple mechanisms (136). They can strengthen intestinal barrier function, including the physical barrier and mucus layer; regulate immune responses, such as by promoting IgA secretion, reducing inflammatory cytokines, and enhancing NK/CD8 T-cell function; and produce beneficial metabolites such as SCFAs, which provide energy for intestinal epithelial cells and exert anti-inflammatory, antioxidant, and pro-apoptotic effects (137). Prebiotics indirectly improve intestinal microecology by providing substrates for probiotics, such as inulin, fructooligosaccharides, and resistant starch, thereby promoting their proliferation.

Animal and human studies indicate that Lactobacillus casei can stimulate immune responses and inhibit tumor development, showing positive effects in preventing recurrence of colorectal cancer and bladder cancer (138). Lactobacillus and Bifidobacterium may have protective effects against colorectal cancer by maintaining microbial balance, reducing carcinogenic enzyme production, and generating antimutagenic organic acids (139). In animal models, combined use of Lactobacillus murinus, Bifidobacterium lactis, and prebiotics can significantly inhibit colon carcinogenesis (140). Under HSD conditions, reductions in Lactobacillus and SCFA levels weaken antitumor immunity, whereas appropriate supplementation with probiotics/prebiotics may help restore microbial balance and metabolite levels, thereby mitigating the adverse effects of HSD on immunity and cancer risk (141).

Probiotics and prebiotics have shown favorable safety and potential efficacy in cancer prevention and treatment. However, their clinical translation remains limited by strain-level differences, interindividual microbiota diversity, and insufficient persistence of colonization. Future randomized controlled trials are needed to define optimal strain combinations, dosages, and application settings, thereby supporting their broader use in cancer prevention and control.

### Emerging microbiota-based therapies

Studies show bidirectional regulation between host miRNAs and the gut microbiota. Host miRNAs can enter bacteria and specifically regulate their growth and gene expression. Conversely, the gut microbiota can secrete extracellular vesicles (OMVs) that deliver miRNAs or other molecules to the host immune system or tumor cells, thereby influencing cancer development, immune escape, and the TME (133, 142).

On the basis of this mechanism, miRNAs have been viewed as potential targets for precision therapy. For example, miRNA mimics or inhibitors may reshape gut microbiota structure and metabolic pathways, improve immune responses, and thereby contribute to cancer prevention and treatment.

Bacteria-derived OMVs are nanoscale vesicles with distinctive advantages in cancer immunotherapy: they can accumulate in tumor tissues, induce IFN-γ production, and activate antitumor responses (143). Through genetic modification, OMVs can carry specific antigens or functional molecules, such as antibodies targeting CTLA-4 or PD-1, to enhance the efficacy of immune checkpoint inhibitors (143, 144). These emerging strategies provide new avenues for integrating gut microbiota modulation with cancer therapy (145). However, substantial interindividual differences in microbiota mean that precision intervention requires large-scale clinical data analysis to identify cancer-associated microbial biomarkers and develop individualized approaches. At the same time, insufficient miRNA targeting specificity, delivery efficiency, and stability, together with OMV immunogenicity and scalable manufacturing, remain major barriers to clinical translation.

In summary, miRNAs and OMVs provide innovative tools for gut microbiota modulation and have broad application prospects in cancer immunotherapy. However, their clinical use remains at an early exploratory stage. Substantial advances in delivery-system optimization, safety validation, and large-scale clinical trials will be required before they can enter routine clinical practice.

## Conclusion and future outlook

The relationship among HSD, the gut microbiota, immunity, and cancer represents a complex and context-dependent network. HSD can directly regulate immune-cell function and indirectly affect the immune system by reshaping gut microbiota composition and microbial metabolites. In some settings, HSD may enhance antitumor immune activity, whereas chronic exposure may promote inflammation, immune exhaustion, microbial dysbiosis, and carcinogenic risk.

Current intervention strategies mainly include salt reduction, probiotics or prebiotics, and emerging microbiota-based approaches such as miRNAs and OMVs. These strategies may reduce cancer risk or improve antitumor immunity by restoring microbial balance and modulating immune responses. However, their effects are likely to depend on host background, baseline microbiota composition, cancer type, and intervention timing.

Future studies should integrate metagenomics, metabolomics, transcriptomics, dietary assessment, and immune profiling to clarify how HSD reshapes the gut microbiota-immunity-cancer network. Longitudinal cohorts, multicenter population studies, and randomized controlled trials are needed to verify causality, evaluate safety, and identify responsive populations. Particular attention should be given to vulnerable groups, including older adults, individuals with obesity, and patients with impaired immune function.

In conclusion, the HSD-gut microbiota-immune axis provides a useful framework for understanding the links between dietary salt intake and cancer biology. A deeper understanding of this axis may support microbiota-informed strategies for cancer prevention, immunotherapy optimization, and personalized dietary intervention (**Figure 6**).

**Figure 6:**
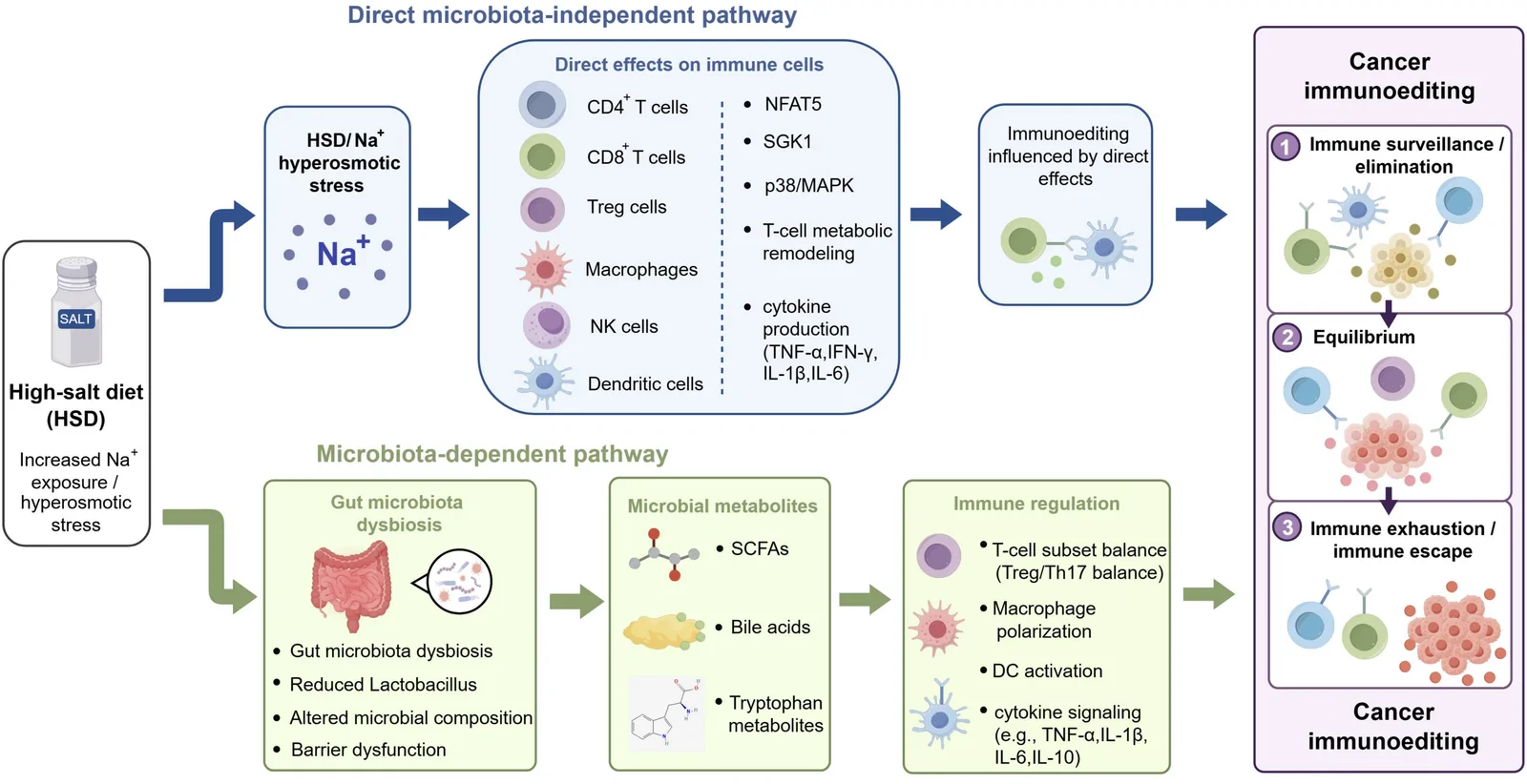
Influence of HSD on the gut microbiota-immunity-cancer network. This image integrates the review framework by connecting dietary salt exposure, microbial dysbiosis, metabolite changes, immune remodeling, and cancer progression or therapy response. It also summarizes the rationale for future interventions targeting diet, probiotics or prebiotics, microbial metabolites, extracellular vesicles, and multi-omics-guided clinical validation.
